# Atopic skin inflammation promotes systemic anaphylactic responses via IL-13 signaling in conventional dendritic cells

**DOI:** 10.21203/rs.3.rs-5892170/v1

**Published:** 2025-02-14

**Authors:** Yasuyo Harada, Takanori Sasaki, Kazushige Obata-Ninomiya, Takahiro Matsuyama, Satoshi Ueha, Shigeyuki Shichino, Takashi Watanabe, Wu Bin, Shuhei Ogawa, Sewon Ki, Yoshie Suzuki, Hideki Ueno, Steven F. Ziegler, Hiromasa Inoue, Peter D. Burrows, Kenneth Murphy, Brian S. Kim, Masato Kubo

**Affiliations:** 1 Division of Molecular Pathology, Research Institute for Biomedical Science, Tokyo University of Science, 2669 Yamazaki, Noda-shi, Chiba 278-0022, Japan; 2 Division of Rheumatology, Department of Internal Medicine, Keio University School of Medicine, Shinanomachi 35, Shinjuku-ku, Tokyo 160-8582, Japan; 3 Benaroya Research Institute, Center for Fundamental Immunology, 1201 Ninth Avenue, Seattle WA 98101-2795; 4 Department of Pulmonary Medicine, Graduate School of Medical and Dental Sciences, Kagoshima University, 1-35-1 Sakuragaoka, Kagoshima 890-0075, Japan; 5 Division of Molecular Regulation of Inflammatory and Immune Diseases, Research Institute for Biomedical Science, Tokyo University of Science, 2669 Yamazaki, Noda-shi, Chiba 278-0022, Japan; 6 Laboratory for Integrative Genomics, Center for Integrative Medical Science (IMS), RIKEN Yokohama Institute, 1-7-22 Suehiro-cho, Tsurumi, Yokohama, Kanagawa 230-0045, Japan; 7 Division of integrated research, Research Institute for Biomedical Science, Tokyo University of Science, 2669 Yamazaki, Noda-shi, Chiba 278-0022, Japan; 8 Laboratory for Cytokine Regulation, Center for Integrative Medical Science (IMS), RIKEN Yokohama Institute, 1-7-22 Suehiro-cho, Tsurumi, Yokohama, Kanagawa 230-0045, Japan; 9 Department of Immunology, Graduate School of Medicine, Kyoto University, Yoshida-Konoe-cho, Sakyo-ku, Kyoto 606-8501, Japan; 10 Department of Microbiology, University of Alabama at Birmingham, Birmingham, AL 35294, USA; 11 Department of Pathology and Immunology, Washington University in St Louis, School of Medicine, St Louis, MO, USA.; 12 Kimberly and Eric J. Waldman Department of Dermatology, Mark Lebwohl Center for Neuroinflammation and Sensation, Marc and Jennifer Lipschultz Precision Immunology Institute, Friedman Brain Institute, Icahn School of Medicine at Mount Sinai, New York, NY 10019, USA; 13 Allen Discovery Center for Neuroimmune Interactions, New York, NY 10019, USA; 14 YH and TS equally contribute to this manuscript as first author.

## Abstract

Cutaneous allergen sensitization (CAS) underlies atopic dermatitis (AD) and leads to various allergic symptoms, including food allergy and anaphylaxis. IL-13 expression by T follicular helper T (T_FH_) has been reported to be involved in generating high-affinity IgE antibodies and causing systemic anaphylaxis.^1, 2^ However, the mechanisms by which IL-13 triggers IgE-mediated allergic responses remain poorly defined. In the present study, we elucidate the role of IL-13 in the CAS-mediated mechanism by which high-affinity IgE antibodies are produced when the same allergen is introduced at a distal site in the secondary sensitization. The CAS model system using mice lacking the cell lineage-specific IL-13 receptor (IL-13R) demonstrated that dendritic cells (DCs), but not T or B cells, are critical in the high-affinity IgE-mediated anaphylactic response. The IL-13 signal in type 2 conventional DCs (cDC2s) enhanced the expression of MHC class II and CD301b, which was essential for the recall of type 2 responses, inducing the production of high-affinity IgE antibodies. Similar IL-13R-expressing DCs were identified in allergic rhinitis and food allergy patients with a history of AD. These findings strongly suggest the importance of DC-specific IL-13 signaling in CAS-induced allergic reactions associated with the atopic march, which is common in human AD patients.

## Introduction

Allergy encompasses a complex array of diseases that combine innate and adaptive immunity, host genetic polymorphisms, and environmental triggers that collectively influence susceptibility to type 2 inflammation. Skin barrier dysfunction in the setting of atopic dermatitis (AD) leads to cutaneous allergen sensitization (CAS).^3^ The atopic march hypothesis proposes that such skin sensitization leads to the subsequent hypersensitivity to allergens at other barrier surfaces, resulting in asthma, food allergy, and its associated anaphylaxis.^4^ The damaged skin barrier in AD allows for the penetration of allergens, which are taken up by Langerhans and dendritic cells (DCs). Skin DCs then migrate to the local lymph nodes and present allergens to T cells, resulting in the generation of type 2 helper T (Th2) and T follicular helper T (T_FH_) cells, which produce the canonical type 2 cytokines interleukin (IL)-4 and IL-13. IL-13 is known to optimally promote the development of allergen-specific IgE, a defining feature of atopy.^5, 6, 7^ Thus, the secondary lymphoid organ represents a key anatomic site for allergen priming. However, the precise mechanisms that convert local allergen sensitization in the skin to systemic allergy remain poorly defined.

Epithelial cell-derived alarmin cytokines such as thymic stromal lymphopoietin (TSLP) and IL-33 act to promote type 2 responses, both innate and adaptive type 2 inflammation in the skin in response to barrier breach and are early initiators of allergen sensitization.^8, 9, 10, 11^ The vitamin D_3_ analog, calcipotriol (MC903), is known to be a potent inducer of TSLP, causing AD-like skin inflammation with barrier dysfunction.^8, 9^ We have also reported that TSLP receptor (TSLPR) expression on CD4 T cells was critical for the survival of Th2 cells.^12^ Analysis of Tslpr^flox/flox^ mice crossed to Zbtb4-cre or Cd4-cre lines has demonstrated that TSLPR signaling in T cells and DCs is vital for Th2-induced inflammation.^13^ In this context, TSLP-treated DCs promote Th2 differentiation in humans and mice by expressing CCL17 and CCL22.^14, 15^ On the other hand, it has been reported that MC903-induced TSLP expression causes T_FH_ development in mouse asthma models.^16^ Langerin^+^ and Langerin^−^ migratory skin DCs contribute to the TSLP-induced T_FH_ differentiation and GC responses, and antigen transport to draining LNs was mainly performed by Langerin^−^ DCs.^17^ Interestingly, TSLP is required for the T_FH_ and Th2 responses initiated by CAS, but not by dermacutaneous allergen sensitization.^18^ Therefore, TSLP expression in the skin caused by CAS is sufficient to induce local allergic reactions. However, how the CAS-mediated local responses lead to systemic IgE responses remains unclear.

In the lung, ILC2-derived IL-13 is necessary for attracting migratory DC2s (migDC2s) to the draining lymph nodes (dLNs) for Th2 cell priming by the upregulation of CCR7.^19, 20^ The migration of migDC2s into dLNs promotes the differentiation of Th2 cells, which express a specific receptor for IL-33. Activated Th2 cells relocalize back to the inflamed lung parenchyma and bronchoalveolar fluid to facilitate chronic airway responses.^21^ Following re-challenge with allergens or IL-33 injection, the Th2 cells attract eosinophils. Interestingly, these Th2-mediated secondary allergic responses were impaired in B cell-deficient μMT mice, in which T_FH_ cell development is defective, implying that T_FH_ cells could be precursors of Th2 cells.^21^ Additionally, it has been reported that steady-state production of IL-13 by skin ILC2s was necessary to facilitate the antigen-presenting ability of CD11b^mid^ migDC2s to favor Th2 differentiation.^22^ However, how IL-13 may contribute to the T_FH_-mediated humoral and memory responses to generate systemic IgE responses remains poorly defined.

A unique subset of IL-13-producing T_FH_ cells, called T_FH_13 cells, has been implicated in the production of anaphylactic high-affinity IgE Abs to inhaled allergens.^1^ We have also reported that Th2 cells could differentiate into GATA3^+^ T_FH_2 cells upon secondary antigen stimulation.^2^ Since activated B cells, including GC-B cells, express IL-13Rα1,^23, 24^ it was speculated that IL-13Rα1^hi^-expressing IgG1 GC-B cells may be the primary target of the T_FH_13-derived IL-13 to induce sequential class-switching to IgE.^1, 2^ On the other hand, IL-13 derived from ILC2s contributes to the IgE responses controlled by T_FH_ cells in response to peanut allergens via CD11c^+^ MHCII^hi^ DCs.^25^ Therefore, how IL-13 contributes to IgE responses and high-affinity IgE production remains unclear.

The IL-4/STAT6 axis is the critical pathway for IgE responses, as genetic deletion of *Il4* or *Stat6* eliminates both total and antigen-specific IgE responses.^26, 27^ The IL-4 derived from T_FH_ is critical for the production of allergen-specific IgE Abs in the GC response.^28^ In contrast, the role of IL-13 in IgE production remains controversial. Previous studies indicated that IL-13-deficient mice showed a selective reduction of IgE antibodies in an OVA immunization model but not in an infection model with the helminth Nippostrongylus.^29, 30, 31^ In contrast, the monoclonal Ab biologic lebrikizumab, which targets human IL-13, has shown a pivotal therapeutic effect in moderate to severe forms of AD as well as inhibiting IgE responses in adults.^32, 33^ Moreover, antigen exposure through the airways without adjuvant induced an IL-13-dependent T_FH_ response in the IgE response to peanut allergens.^16^ However, how IL-13 leads the allergen-specific IgE response independent of IL-4 is still unclear.

The IL-13 signal is transduced by the heterodimeric complex of the IL-13 receptor α chain 1 (IL-13Rα1) and IL-4Rα. The IL-13 signal results in activation of JAK1, JAK2, and tyrosine kinase 2 (TYK2) and subsequent phosphorylation of STAT3 and STAT6.^34, 35^ In human monocytes/macrophages, it was reported that IL-13 elicits Jak2-STAT3 and Jak1-STAT1 pathways.^36^ IL-4-activated STAT6 controls IgE class-switching in both human and mouse B cells, but there is no evidence to show a contribution of IL-13. These observations suggest that IL-13 controls IgE antibody (Ab) responses independently from IL-4 and that IL-13 binding to the cognate receptor provides an independent signal in different cell lineages.

In the present study, we show that cutaneous antigen sensitization via AD-like skin inflammation causes hyperactivation of Th2 cells in the dLNs, which subsequently triggers high-affinity IgE responses that cause anaphylaxis when the same T-dependent antigen is introduced at distal sites. Our series of analyses indicated that the specific activation of the cDC2 subset by IL-13 signal promoted the efficient generation of Th2 memory cells and T_FH2_ cells, which induce the production of allergen-specific IgE and high-affinity IgE antibodies. Therefore, IL-13-dependent cDC2 activation is a critical pathway controlling CAS-mediated allergic responses.

## Results

### CAS induces a high-affinity IgE response and IL-13-expressing T_FH_ cells

**Figure 1.**
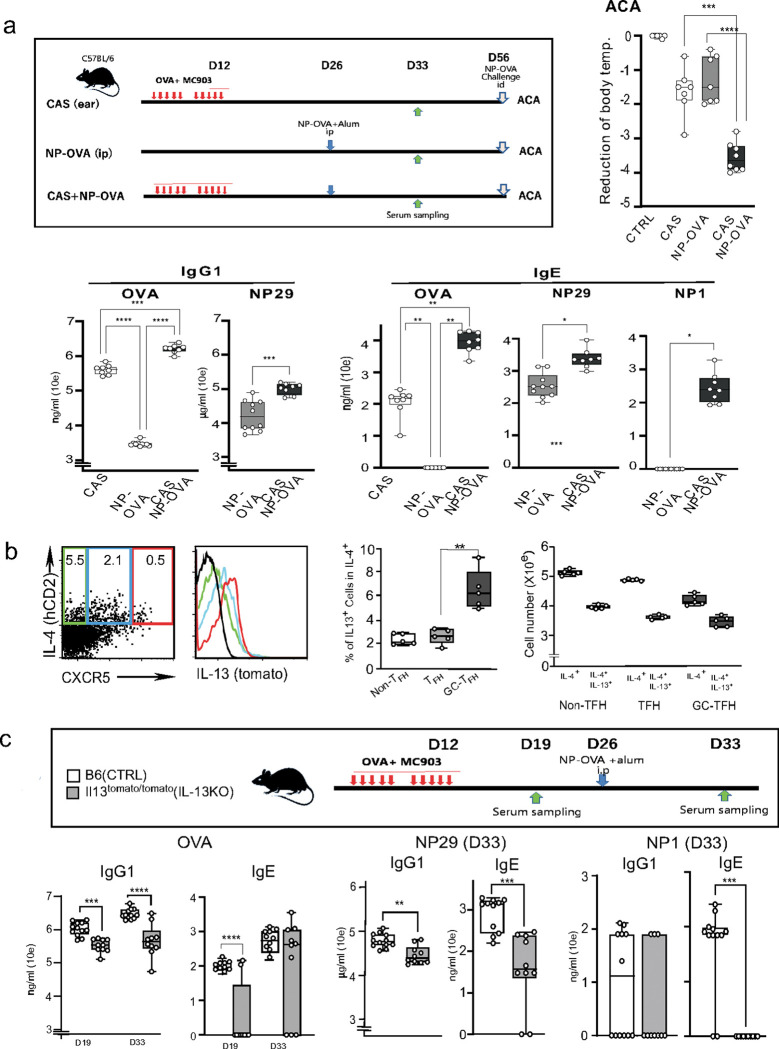
Cutaneous allergen sensitization (CAS) treatment with MC903 induces high-affinity IgE and IL-13-expressing T_FH_ cells. (**a**) The upper left panel depicts the three experimental groups. CAS group: C57BL/6J mice were treated with the irritant MC903 (2mM)+OVA (100μg) by daily topical application to both ears for ten days. NP-OVA group: Mice were intraperitoneally immunized with NP16-OVA (100μg) in an alum adjuvant. CAS+NP-OVA group: Mice were treated with the MC903+OVA and were further immunized i.p. with NP16-OVA at D26. To assess the active cutaneous anaphylaxis (ACA) response, the CAS-treated mice were challenged intradermally with NP16-OVA at D56 and their body temperature was measured (upper right) ^23^. The lower panels indicate IgG1 and IgE responses to OVA, NP29, and NP1 in the three groups at D33 (n=10). (**b**) **IL-13 expression by T**_**FH**_ **cells.** hCD2-IL-4 Bac TgX Il13^tomato^ KI mice were treated with CAS+NP-OVA and examined for IL-4 (hCD2) and IL-13 (tomato) expression by CXCR5^+^ T_FH_ cells on D33. The figure shows the IL-13 expression in CXCR5^−^ (nonT_FH_), CXCR5^mid^ (T_FH_), and CXCR5^hi^ (GC-T_FH_) fractions (n=5). The middle panel shows the percentage of IL-13^+^ cells among IL-4^+^ T_FH_ cells. The right panel indicates the number of IL-4^+^ cells and IL-4 and IL-13 double-positive cells. (**c**) **Role of IL-13 in IgG1 and IgE responses.** Top: the experimental setup. The bottom panels show IgG1 and IgE Ab titers against OVA(D19), NP29, and NP1(D33) of CTRL and Il13^tomato/tomato^ (*Il13* KO) mice (n=10). Data are shown as mean ± SD, unpaired *t*-test, and P values are indicated as * p<0.05, ** p<0.01, *** p<0.001, **** p<0.0001.

Antigen sensitization in chronically damaged skin induced by topical application of MC903 leads to a break in tolerance, manifesting as a severe allergic reaction upon repriming with appropriate antigens. We applied topical MC903 daily along with the model antigen OVA, allowing proximity between the antigen and the damaged skin. The MC903-treated mice showed robust production of OVA-specific IgG1 and IgE (Fig.1a). To monitor the impact of CAS on the response to an inappropriate allergen by systemic antigen exposure, we further immunized the mice intraperitoneally with 4-hydroxy-3-nitrophenyl acetyl (NP)-OVA in alum adjuvant. In this case, the hapten NP is a primary antigen that B cells encounter for the first time. The NP-OVA immunization promoted about a 10-fold increase in OVA responses and 10- or 100-fold higher levels of anti-NP IgG1 or IgE antibodies than those seen following systemic immunization alone (Fig.1a). Moreover, the anti-NP IgE, but not IgG1, antibodies included high-affinity Abs recognizing NP1, but such high-affinity IgE did not appear with NP-OVA priming alone. These results indicated that the CAS-induced OVA-specific memory T cells could induce high-affinity anti-NP IgE Abs even upon primary B cell activation. Furthermore, the intradermal challenge with NP-OVA at the unlinked site caused severe anaphylaxis symptoms (Fig.1a), indicating that the high-affinity anti-NP IgE Abs were initiated by the CAS-induced OVA-specific memory T cells even when OVA was introduced at distal sites. The high-affinity IgE correlated with the severity of anaphylaxis.

IL-13 secreted by T_FH_13 cells plays a critical role in the high-affinity IgE response.^1, 2^ We first asked whether CAS induced the development of T_FH_13 cells. IL-4 and IL-13 double-reporter mice^37^ were treated with CAS and systemic immunization with NP-OVA, and we then compared IL-13 expression by CXCR5^−^ (Th2), CXCR5^mid^ (preT_FH_), and CXCR5^+^ T_FH_ cells among the IL-4^+^ CD4 T cells. The CXCR5^+^ T_FH_ cells were the most abundant population that highly expressed both IL-4 and IL-13 (Fig.1b). CAS-treated IL-13-deficient mice (IL-13KO), in which tomato reporter genes were knocked in at the *Il13* locus on both alleles, ^37^ exhibited a significant reduction in the anti-NP29 IgG1 and IgE response (Fig.1c). The IL-13KO mice further showed a striking decrease in anti-NP1 IgE but not IgG1 Abs (Fig.1c). These results indicated that CAS played a role in the generation of T_FH_13 cells, which may control the induction of high-affinity IgE Abs.

### IL-13 signaling in dendritic cells selectively contributes to the skin sensitization-mediated IgE responses.

**Figure 2.**
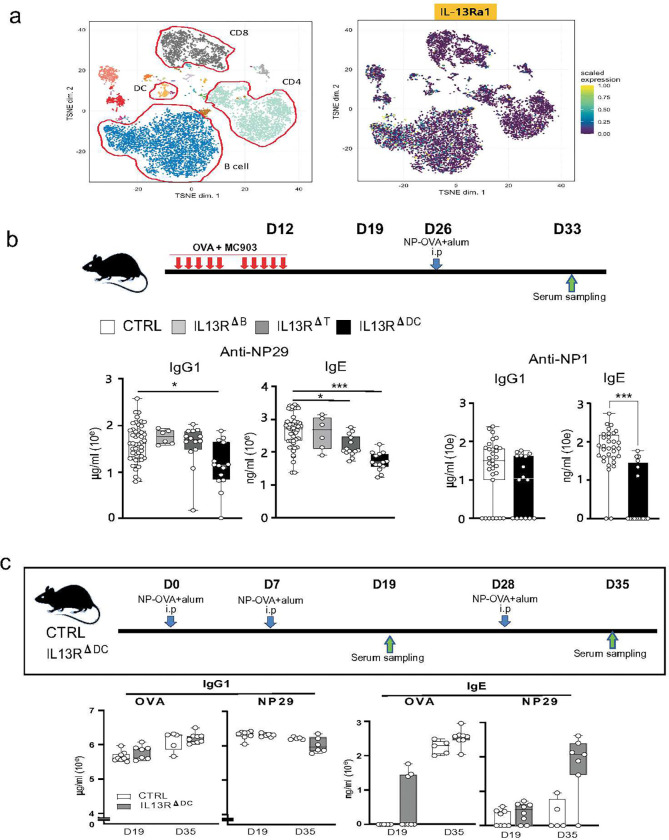
The expression and function of IL-13Ra1 by T, B, and dendritic cells. **(a) The expression of IL-13Ra1 was analyzed in T and B cells and DCs.** Cells obtained from the spleen, LN, and PBMC of unprimed mice were stained with Maxpar metal-conjugated 34 antibodies (Maxpar OnDemand Mouse Immune Profiling Panel Kit). The clusters outlined in red indicate CD4 and CD8 T cells, B cells, and DCs (left), and the IL-13Ra1+ cells were visualized by CyTOF analysis (right). (**b**) **Role of the IL-13 signal in skin sensitization.** Control (CTRL, n=58), *Il13ra1*^f/f^*Cd4*-*Cre* (*Il13ra1*^ΔT^, n=14), *Il13ra1Cd79a*-*Cre* (*Il13ra1*^ΔB^, n=7) and *Il13ra1*^f/f^*Cd111c*-*Cre* (*Il13ra1*^ΔDC^, n=25) mice were treated with CAS+NP-OVA as described in Fig.1a. Anti-NP29 and anti-NP1 IgG1 and IgE titers were measured by ELISA at D33. (**c**) **Role of the IL-13 signal in systemic responses.** CTRL (n=7) and *Il13ra1*^ΔDC^ (n=7) were i.p. immunized with NP16-OVA in alum adjuvant at D0 and D7 and boosted at D28. Anti-OVA and anti-NP29 IgG1 and IgE titers were measured by ELISA at D10 and D35. Data are shown as mean ± SD, unpaired *t*-test, and P values are indicated as * p<0.05, ** p<0.01, *** p<0.001.

T_FH_-derived IL-13 contributes to controlling sequential class-switching to IgE.^1, 38^ However, whether IL-13 acts directly on GC-B cells or indirectly via another cellular target is unclear. Therefore, we asked which immune cells mainly express the receptor for IL-13 (IL-13Ra1). We analyzed the expression of IL-13Ra1 by mass-cytometry (CyTOF) analysis of the cervical LN cells from mice treated with CAS and systemic immunization and found that it was dominant in B cells but was also significantly expressed by CD4 T and DCs (Fig.2a).

To further identify the primary target of the IL-13 signal in CAS and systemic immunization, we generated mice containing *lox*P-flanked *Il13ra1* alleles (*Il13ra1*^f/f^) (**S-Fig.1a&b**) and crossed them to mice expressing Cre recombinase under the control of the T-cell-specific *Cd4* promoter (*Il13ra1*^f/f^*Cd4*-*Cre*; referred to as *Il13ra1*^ΔT^), the B-cell-specific promoter of the gene encoding the BCR signaling subunit CD79A (*Il13ra1Cd79a*-*Cre*; referred to as *Il13ra1*^ΔB^) or the CD11c promoter (*Il13ra1*^f/f^*Cd111c*-*Cre*; referred to as *Il13ra1*^ΔDC^). After CAS and systemic immunization, we measured anti-NP IgG1 and IgE titers on day 33 (D33). We expected that B cells would be a direct target of IL-13 because B cells showed the most abundant expression of Il13ra1 transcript (**S-Fig.1c)**. However, there was no significant reduction in the IgG1 and IgE responses to NP29 in the *Il13ra1*^ΔB^ mice (Fig.2b). The *Il13ra1*^ΔT^ mice showed a subtle but significant reduction in the IgE response to NP29 but no reduction in the IgG1 responses to NP29. In contrast, *Il13ra1*^ΔDC^ mice showed a marked reduction in the anti-NP29 IgG1 and IgE and the anti-NP1 IgE responses. The DC-specific deletion consistently showed selective loss of transcript and cell surface expression of IL-13Ra1 in CD11c DCs but not in T and B cells (**S-Fig.1 c&d**). Interestingly, the IL-13 signaling in DCs has a selective role in type 2 humoral responses, in which allergens primarily enter through the skin. The *Il13ra1*^ΔDC^ mice treated with systemic NP-OVA immunization without CAS treatment showed no reduction in the anti-OVA and anti-NP IgG1 after boosting (D35) (Fig.2c). Systemic immunization induced detectable levels of anti-OVA IgE and no or subtle anti-NP IgE antibodies in both control and *Il13ra1*^ΔDC^ mice. We also found a similar pattern following immunization with the SARS-CoV-2 spike protein (**S-Fig.2**). These findings suggest that DCs are the primary target of IL-13 in generating type 2 humoral responses, including high-affinity IgE antibodies. The IL-13-mediated type 2 humoral responses are selectively associated with initial allergen priming in the skin.

### Skin sensitization promoted the accumulation of IL-13Ra1^+^ cDC2s in dLN and spleen.

**Figure 3.**
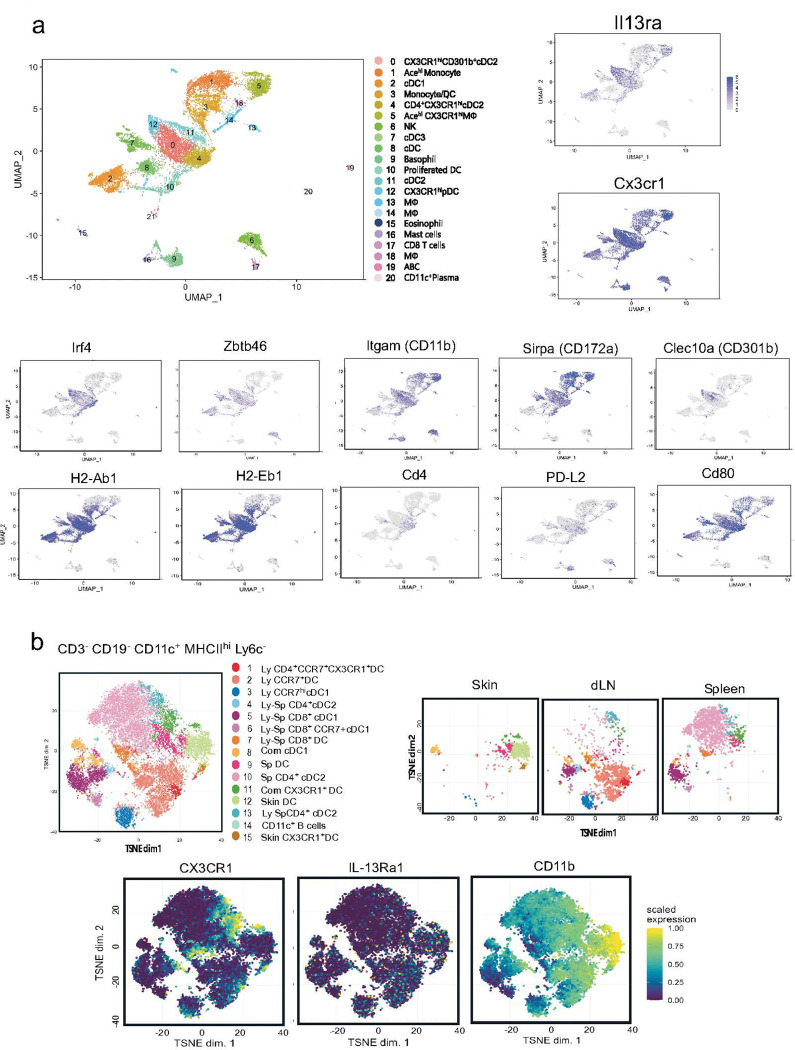
CyTOF and scRNAseq analysis of CD11c^+^ cells. **(a) scRNAseq analysis of CD11c^+^ cells in the inflamed spleen.** Single CD11^+^ cells (*n*=14802) were isolated after eliminating the lineage^+^ cells (CD3, B220, NK1.1, and TER119) from the spleen of CAS+NP-OVA treated mice and scRNAseq analysis was performed. An unsupervised Uniform Manifold Approximation and Projection (UMAP) display of cell clustering based on gene expression (left). The Cluster indexes 0 to 20 indicate subset identification and the tissue distribution of the cDC subsets. The UMAP algorithm identified 20 clusters, including eight DC clusters. Clusters expressing *Il13ra1* and *Cx3cr1* are illustrated (right). Clusters expressing cDC2 markers (*Irf4*, *Zbtb46*, *Itgam, Sirpa, Clec10a, MHC2, Cd4, Pdcd1lg2*, and *CD80*) are illustrated (bottom). (**b**) **t-SNE plots for 34 immune markers from CyTOF analysis of CD11c**^**+**^ **cells**. Cells were obtained from the skin, dLNs, and spleen of unprimed mice (n=2) and were stained with Maxpar metal-conjugated antibodies for CyTOF analysis. The CD3^−^ CD19^−^ Ly6G^−^ CD11c^+^ MHCII^+^ cells were selected from the whole data set and displayed with a t-SNE algorithm (Top left). The t-SNE algorithm identified 14 DC clusters. The top right panels show the tissue distribution of the cDC subsets. The bottom panels show the heat map profile of the cell surface expression of IL-13Ra1, CX3CR1, and CD11b in the mixture of three tissues.

The above results indicated a specific role of the IL-13 signal in DC for CAS-mediated allergic responses. Previous evidence suggested that IL-13 modified the DC character to activate Th2 responses.^(42*,*43)^ To identify the DC population responsible for the CAS-mediated allergic responses, we analyzed the expression of the *Il13ra1* transcript by single-cell RNA sequencing (scRNAseq) of CD11c^+^ cells in the inflamed spleen after priming with the combination of CAS and systemic immunization (D33). An unsupervised Uniform Manifold Approximation and Projection (UMAP) algorithm identified 20 clusters, including six cDC clusters (Fig.3b). The cDCs were further subdivided into cDC1s (Irf8^+^ Xcr1^+^, Cd81^+^, Batf3^+^, GCsam^+^, Tcf4^+^), cDC2s (Sirpa^+^, S100a4^+^, Tcf4^+^, Klf4^+^, Irf4^+^) and cDC3s (Ccr7^+^, Nudt17^+^). Four clusters, C0, C4, and C11 contained cDC2-specific gene signatures. The *Il13ra1* transcript was expressed in Ccr9^+^, Tcf4^+^, Siglec^+^pDC (C12), cDC2s (C0 and C11) and macrophages (C5). The *Il13ra1* transcript was robustly expressed in C0 and C12. Several cDC2 transcriptional signatures, *Zbtb46*, *Tcf4*, *Klf4*, and *Irf4* were abundant in C0. Interestingly, this cluster also expressed *Cx3cr1.* Furthermore, C0 also expressed several cDC2 marker genes, *Sirpa* (CD172a), *Bst2*, *Clec10a* (CD301b), and *Pdcd1g 2* (PDL2) and highly expressed *MHC2*, *Itgam* (CD11b) and several activation markers, *Cd80*, Cd86, *Icosl*, *Tnfsf* (OX40L), *Clec7a* (Dectin-1), and *Cd209a* (CD-SIGN) (Fig.3b & **S-Fig.3**). These results indicate that in the inflamed spleen, IL-13Ra1 was predominantly expressed by CX3CR1^+^ cDC2s.

We further performed CyTOF analysis to examine cell surface expression of IL-13Ra1 on the MHCII^+^ CD11c^+^ population in independent tissues, skin, and cervical LNs, which are proximal to the initial priming site and the spleen, which is proximal to the secondary priming site. An unsupervised t-Distributed Stochastic Neighbor Embedding (t-SNE) algorithm^39^ identified 14 DC subsets among the three tissues we tested (Fig.3b). The DC populations in steady state (no stimuli; NS) were classified into tissue-specific subsets: the skin contained two dermal specific DCs, CX3CR1^+^ DC and CD11b^hi^ F4.80^+^DC,^22^ cervical LNs comprised four Lymphatic (Ly) subsets, CD4^+^CCR7^+^CX3CR1^+^DC (C1), CCR7^+^DC (C2), CD4^+^cDC2 (C4) and CCR7^hi^ cDC1 (C3), spleen composed of two splenic subsets, splenic DC (Sp DC, C9) and CD4^+^ cDC2 (C10) (Fig.3b). There were six shared populations: three CD8^+^cDC1 (C5, 6 and 7) and C4 were shared by LN and spleen, cDC1 and CX3CR1^+^ DC were found in all three tissues (Com cDC1; C8, Com CX3CR1^+^ DC; C11). Cell surface expression of IL-13Ra1 was found in two skin DC subsets (C12 and C15), C1, C2, C4, C9, C10, and C11. C1, C11, and C15 highly expressed CX3CR1, a membrane-bound receptor for the fractalkine CX3CL1, widely expressed by monocytes, macrophages, and monocyte-derived DCs.^40, 41, 42, 43^ These results suggested that CX3CR1^+^DCs were migratory, contributing to antigen delivery.

**Figure 4.**
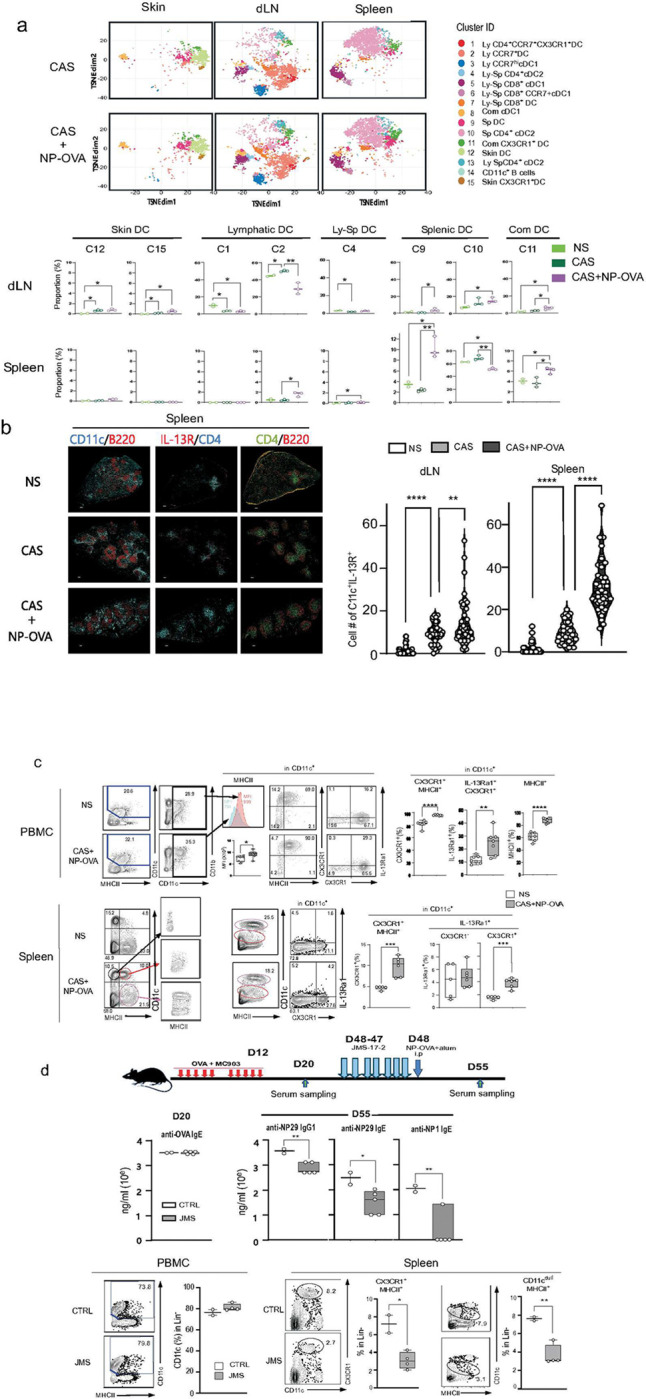
Tissue dynamics of cCD2s in the CAS-treated mice. **(a) t-SNE plots for 34 immune markers from CyTOF analysis of CD11c^+^ cells.** Cells were obtained from the skin, dLNs, and spleen of unprimed (NS)(n=2), CAS (n=3), and CAS+NP-OVA (n=3) mice. The top panel showed the t-SNE algorithm of the CD3^−^ CD19^−^ Ly6C^−^ Ly6G^−^ CD11c^+^ MHCII^+^ cells in NS, CAS, and CAS+NP-OVA treated mice. The bottom panel showed the proportion of each DC cluster in the skin, dLN, and spleen of three priming situations, NS, CAS, CAS+NP-OVA. (**b**) **Localization of IL-13R+ DCs at the T-B border** Immunohistochemical staining was performed on sections from the spleen of the NS (n=3), CAS (n=3), and CAS+NP-OVA (n=3) mice. The spleen sections were stained with CD11c-Alexa647/B220-PE (left), IL-13Ra1 (HM1140-biotin/avidin-PE)/CD4-Alex488 (middle), and CD4-Alex488/B220-PE (right) (X4). The right panel shows the number of the CD11c/IL-13Ra1 double-positive cells present in 10 fields at 20x for each of three priming situations, NS, CAS, CAS+NP-OVA in dLN and spleen. (**c**) **Combining CAS and systemic immunization increased IL-13R**^**+**^ **DCs in PBMC and the spleen.** The upper panel represents PBMC. Cells obtained from NS (n=7) and CAS+NP-OVA (n=7) mice were stained with CD3, B220, MHCII, CD11c, CD11b, CX3CR1 and IL-13Ra1, and the data displayed the lineage^−^ cells (CD3^−^ B220^−^). The upper left shows representative flow cytometry, and the upper right shows the mean ± SD of the percentage of CX3CR1^+^MHCII^+^, IL-13Ra1^+^ CX3CR1^+^ and MHCII^+^ fractions in CD11c^+^DCs. The bottom panel represents spleen cells obtained from NS (n=5) and CAS+NP-OVA (n=6) mice. The bottom left panel shows representative flow cytometry and the right panel shows the mean ± SD of the % of CX3CR1^+^MHCII^+^ and CX3CR1^−^IL-13Ra1^+^ and CX3CR1^+^IL-13Ra1^+^ in CD11c^+^DCs. (**d**) **A CX3CR1 antagonist blocks attraction of CX3CR1**^**+**^**MHCII**^**+**^**DCs to spleen.** The upper panel represents the experimental design. CAS-administered CTRL (n=2) and JMS (n=5) mice were treated i.p with 0.2mg/mouse of JMS for seven days before i.p immunization with NP16-OVA. The middle shows anti-OVA IgE titers on D20 (left) and anti-NP29 and anti-NP1 IgG1 and IgE titers on D55 (lower panel). Cells were stained with MHCII, CD11c and CX3CR1 and the data displayed the lineage^−^ cells (CD3^−^ B220^−^). Data are shown as mean ± SD, unpaired *t*-test, and P values are indicated as * p<0.05, ** p<0.01, *** p<0.001, **** p<0.0001.

The above results suggested that the in vivo dynamism of IL-13Ra1^+^DCs would be the critical link between the flamed dLNs and the spleen. To examine this possibility, we performed a comprehensive CyTOF analysis to study the tissue dynamism of 14 distinct DC subsets in NS, local skin priming (CAS), and the combination of CAS and systemic immunization (CAS+NP-OVA) (Fig.3b & 4a). Following CAS treatment, the cell number in the cervical LNs increased at D19, while the proportions of C1 and C4 deceased. In contrast, the major subset in the dLN, C2, showed a significant increase (Fig.4a). In this condition, skin DC C12 and C15 - the subsets expressing IL-13Ra1 in the steady state - increased in the D19 dLNs, suggesting that the IL-13R+ skin DC migrate into dLNs around the CAS site. Systemic immunization with NP-OVA increased C9 and C11 in the spleen, where the allergen entered. The major subset in the spleen, C10, was significantly decreased, while the C9, C10 and C11 subsets increased in dLNs (Fig.4a). In addition, IL-13Ra1 was detected in C2 and C1 of the flamed dLNs, and C11 and C9 of the flamed spleen (Fig.3b). These results indicated that IL-13Ra1 is co-expressed with CX3CR1 in the neighboring SLO of the allergen entry.

**Figure 5.**
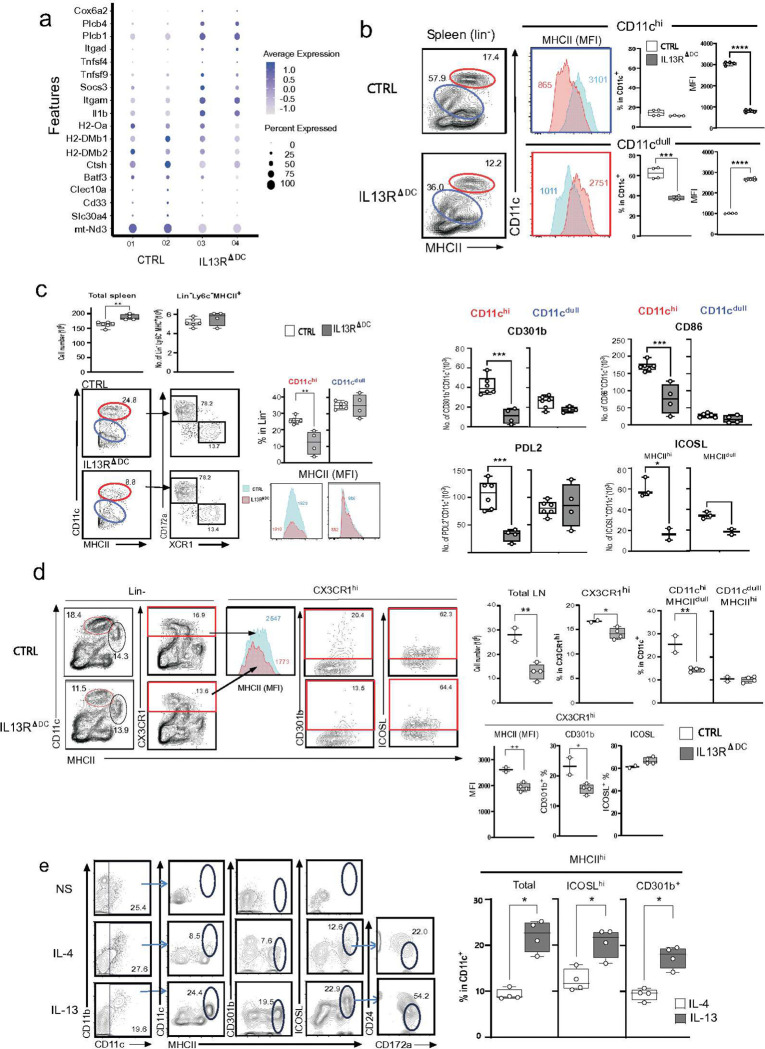
The IL-13 signal controls the expression of MHCII and ICOSL in DCs. **(a) Enrichment analysis of DEGs in CTRL and *Il13ra1*^ΔDC^ mice.** RNA-Seq analysis of CD11c^+^ cells was performed in CTRL and *Il13ra1*^ΔDC^ mice as described in Fig.3a. Comparison of the C0 cDC2 clusters in scRNAseq analysis between CTRL (n=2) and Il13ra1ΔDC (n=2) mice identified 115 DEGs. The color bar indicates expression levels, and the circle size indicates the percentage of expression in two individual mice of CTRL (01 and 02) and *Il13ra1*^ΔDC^ (03 and 04). (**b**) **MHCII expression in unprimed splenic DCs.** Spleen cells obtained from CTRL (n=4) and Il13ra1^ΔDC^ (n=4) unprimed mice and MHCII expression in CD11c^hi^ and CD11c^dull^ was analyzed as described in Fig.4c. The left panel shows representative flow cytometry and the right panel shows the mean ± SD of mean fluorescence intensity (MFI) of MHCII and the percentage of CD11c^hi^ and CD11c^dull^ populations in the spleen. **(c) The impact of the IL-13 signal on CD11**^**hi**^ **DCs in the inflamed spleen.** The spleen was obtained from CTRL (n=6) and *Il13ra1*^ΔDC^ (n=4) mice treated with CAS+NP-OVA (D33). The upper panel shows the number of total cells and MHCII^+^ DCs (Lin^**−**^Ly6C^**−**^). Cells were stained as described in Fig.4c to examine MHCII MFI and the percentage of CD11c^hi^ and CD11c^dull^ populations. The bottom left shows representative flow cytometry data of CTRL and Il13ra1^ΔDC^. The right panel shows the cell numbers of total CD301b, CD86, PDL2 and ICOSL in CD11c^hi^ or CD11c^dull^ population. (**d**) **The impact of the IL-13 signal on CAS priming.** Cells were obtained from the dLN of CTRL (n=2) and *Il13ra1*^ΔDC^ (n=4) mice treated with CAS (D19). The linage^**−**^ DCs were stained with CD11c, MHCII, CX3CR1, CD301b and ICOSL. The left panel shows representative data to examine MHCII MFI in CX3CR1hi DCs and the percentage of CD11c^hi^ MHC^dull^ and CD11c^dull^ MHC^hi^ populations. The upper right panel shows the mean ± SD of the total cell number of dLN and the percentage of CX3CR1hi, CD11chiMHCIIdull, and CD11c^dull^MHCII^hi^. The bottom right panel shows a comparison of the MHCII MFI and the percentage of CD301b^+^ and ICOSL^+^ in CX3CR1^hi^ DCs. (**e**) **The *in vitro* impact of IL-4 and IL-13 on PBDC.** The CX3CR1^+^PBDCs were prepared from PBMC and cultured with IL-4 or IL-13 (1–10μg/ml) for 24 hours, and then expression of MHCII, CD301b, ICOSL and cDC2 markers (CD24 and CD172a) was examined. The left panel shows the representative data, and the right panel shows the mean ± SD of the percentage of total, CD301b^+^ and ICOSL^+^ cells among CD11c^+^ cells. Unpaired *t*-test and P values are indicated as **** p<0.0001, *** p<0.001, ** p<0.01, * p<0.05.

Consistently, immunohistological staining indicated the emergence of the IL-13R^+^ DCs in the dLN and the spleen following CAS+NP-OVA treatment. The number of IL-13Ra1^+^ DCs significantly increased in the CAS+NP-OVA group compared to unprimed mice, with a marked increase compared to CAS alone (Fig.4b). The IL-13R DCs were predominantly localized in the T cell zone, the T-B border area, and the interfollicular regions (Fig.4b, **S-Fig.5a**). Notably, IL-13Ra1 expression overlapped well with CX3CR1 and CD301b (**S-Fig.5a&b)**, which is consistent with the scRNAseq results (Fig.3a). Taken together, these results indicated that IL-13R DCs preferentially migrated and localized in the T-B border area and the interfollicular regions where they contribute to T cell activation in the spleen.

**Figure 6.**
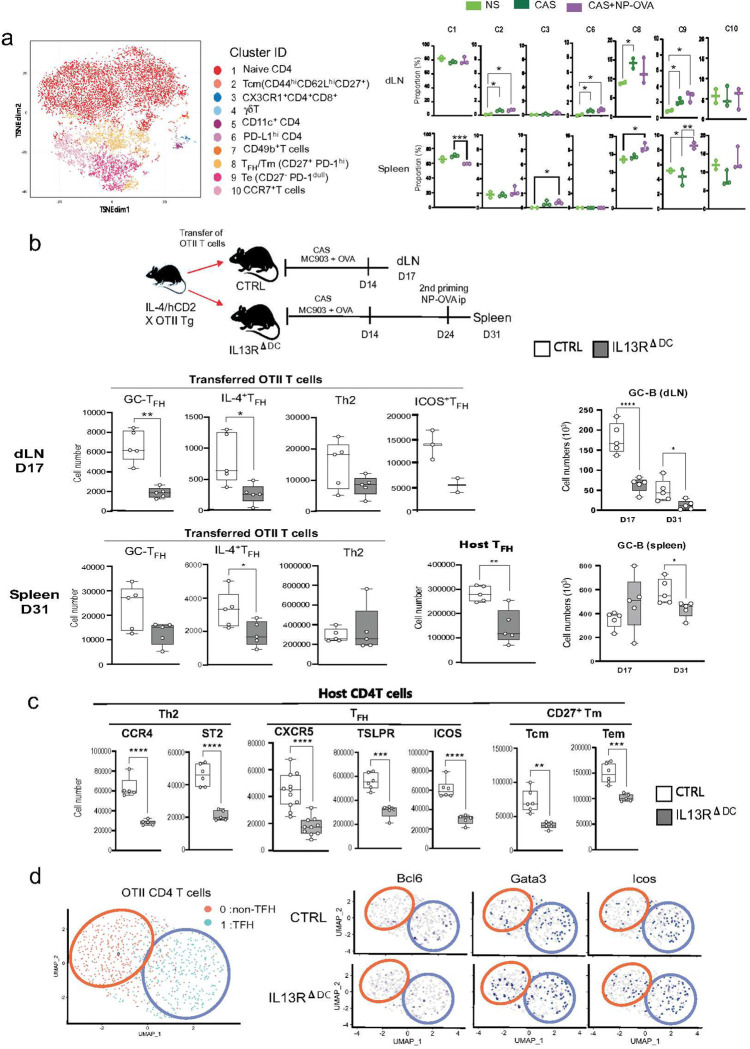
The IL-13 signal in DCs supports T_H_2 and T_FH_ responses. ** (a) CD4 T cell dynamics in skin sensitization.** Cells were obtained from the spleen and dLNs of unprimed (NS, n=2), CAS (n=3), and CAS+NP-OVA (n=3) mice. The CyTOF data of the CD3^+^ CD19^−^ TCRb^+^ TCRgd^−^ CD4^+^ CD8^−^ cells were processed with the t-SNE algorithm. The left panel displays the cluster ID of CD4 T cell subsets. The right panel shows the proportion of each T cell cluster in the skin, dLN, and spleen of the three priming situations, NS, CAS, CAS+NP-OVA. (**b**) **Development of IL-4**^**+**^**Th2 and T**_**FH**_ **cells in the dLNs and spleen.** The top panel shows the design of the transfer experiment. Sorted CD4^+^T cells from the OTII mice crossing with IL-4 reporter (IL-4/hCD2) mice were transferred to CTRL (n=5), and IL-13RΔDC recipient (n=5) mice, and the mice were treated with CAS and CAS+NP-OVA. TCRVB5 positive (transferred OTII) and negative (host) CD4 T cells were isolated from dLN and the spleen at D17 and D31. The upper left panel shows D17 dLN results indicating the cell numbers of GC-T_FH_ (CXCR5^hi^PD-1^+^), IL-4^+^T_FH_ (hCD2^+^CXCR5^+^), Th2 (hCD2^+^CXCR5^−^), and ICOS^+^-T_FH_ (CXCR5^+^ICOS^+^) cells derived from OTII. The lower left panel shows D31 spleen results indicating the cell numbers of GC-T_FH_, IL-4^+^T_FH_, Th2 derived from OTII. The middle panel shows the cell numbers of host T_FH_ (CXCR5^hi^PD-1^+^) cells. The right panel shows the cell numbers of GC-B (GL-7^+^FAS^+^) cells of dLN and the spleen. (**c**) **Development of host Th2, T**_**FH**_**, and memory T cells in dLNs after CAS.** The expression of CCR4, ST2, CXCR5, TSLPR, ICOS, and CD27 on host cells was examined in D17 dLN. The figure shows the cell number of CCR4^+^, ST2^+^, CXCR5^hi^, TSLPR^+,^ and ICOS^+^ in CD4^+^ T cells. Memory status was assessed by CD62L, CD44, and CD27 expression in CD62L^hi^CD44hi (Tcm) and CD62L^−^CD44^hi^ (Tem) CD4 T cells. (**d**) **Transcriptional dynamics of OTII T cells in the IL-13R**^**ΔDC**^ **mice.** TCRVB5^+^ T cells were sorted from D31 spleen cells of the OTII transferred mice as described in (**b**), and scRNAseq analysis was performed. The red circles illustrate cluster 0, indicating non-T_FH_ gene expression. The blue circles illustrate cluster 1, indicating T_FH_ gene expression. The cluster expression profiles of T_FH_ (*Bcl6* and *Icos*) and Th2 (*Gata3*) markers in CTRL and IL-13R^ΔDC^ mice are shown in the right panel. P values by unpaired *t*-test are indicated as * p<0.05, ** p<0.01, *** p<0.001, **** p<0.0001 with unpaired *t*-test.

Interestingly, CX3CR1^+^ DCs were abundant in peripheral blood, and approximately 80% of DCs (CD11c^dull^ CD11b^hi^) were CX3CR1^hi^ even under a steady state. CyTOF analysis of peripheral blood mononuclear cells (PBMC) indicated that this CX3CR1^hi^ peripheral blood DCs (PBDC) corresponds to C11 in Fig.4a (**S-Fig.6**). Following CAS+NP-OVA treatment, the CX3CR1^hi^ PBDC increased to over 90％ with the increased expression of IL-13Ra1^+^DCs and MHCII (Fig.4c). A similar increase in CX3CR1^+^MHC^+^DCs was also observed in the spleen after the CAS+NP-OVA treatment. These expanded DCs exhibited similar phenotypes to PBDCs, CX3CR1^hi^ CD11c^dull^ CD11b^hi^. IL-13Ra1 expression was significantly increased in splenic CX3CR1^hi^ DCs, with no significant change in CX3CR1^lo^ DCs due to more considerable deviation in the steady state (Fig.4c).

Previous reports have shown that CX3CR1^+^DCs migrate to inflamed SLOs through CX3CR1-CX3CL1 interactions.^44^ Therefore, we hypothesized that the CX3CR1^+^ PBDC might play a role in delivering NP-OVA allergen from peripheral blood to the spleen in systemic immunization. To test this possibility, we used the CX3CR1 antagonist, JMS-17–2 (TargetMol), to block the CX3CR1-CX3CL1 interactions. The CAS-treated mice with significant anti-OVA IgE Ab titers were treated with JMS-17–2 (JMS) prior to the systemic NP-OVA priming (Fig.4d). The JMS treatment significantly inhibited anti-NP IgG1 and IgE responses and decreased the proportion of CX3CR1^+^DCs in the inflamed spleen, while slightly increasing CX3CR1^hi^DCs in PBMC (Fig.4d). These results indicated that the CX3CR1+ DCs were required for allergen delivery to the SLO around the secondary priming site.

### The IL-13 signal controlled the expression of MHCII and CD301b on DCs.

The above results indicated that CX3CR1 plays a role in allergen delivery via CX3CR1-CX3CL1 interactions. However, how the IL-13 signal in DCs leads to high-affinity IgE Ab production remains still unclear. Thus, we compared the scRNAseq data between the control- and the IL-13R^ΔDC^- derived splenic DCs. Gene expression indicated that the IL-13R^ΔDC^ DCs had decreased expression of MHC-related genes (H2-Oa and H2-DMb1 and -DMb2) and cDC2 signature genes, *batf3*, and *Clec10a* (CD301b) (Fig.5a). Therefore, the IL-13 signal may regulate the expression of MHCII in DCs. There are three DC populations, CD11c^hi^ MHCII^+^, CD11c^dull^ MHCII^lo^, and CD11c^lo^ MHCII^+^ DCs, in the spleen (Fig.5b). When we compared the MHCII expression between CD11c^hi^ (red) and CD11c^dull&lo^ (blue) DCs, the unprimed IL-13R^ΔDC^ mice exhibited significantly decreased mean fluorescence intensity (MFI) of MHCII on the CD11c^hi^ DCs but not on the CD11c^dull&lo^ DCs (Fig.5b). These results indicated that the CD11c-cre deletion in the IL-13R^ΔDC^ mice affected on the MHCII expression level on CD11c^hi^ DCs

**Figure 7.**
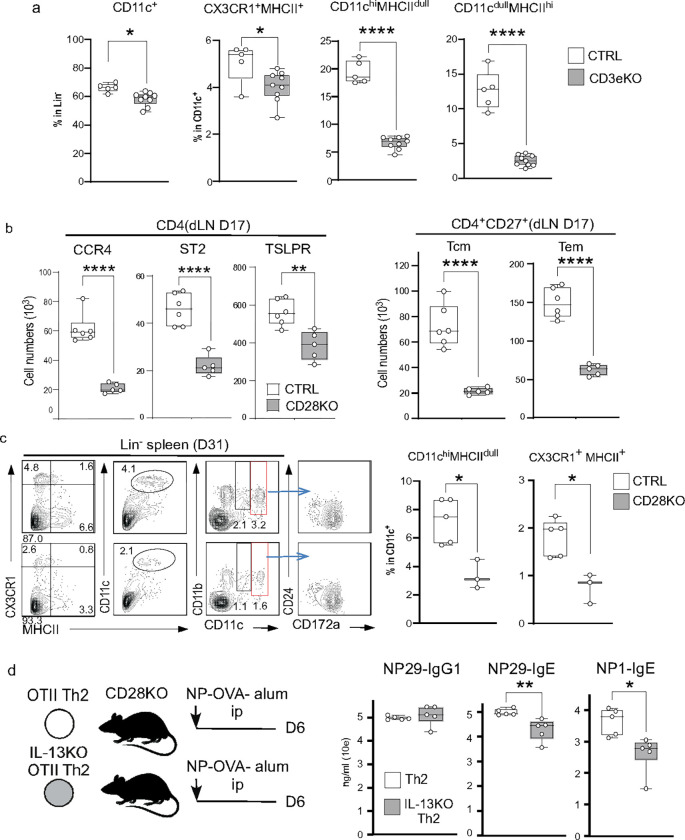
Memory Th2 cells are essential for the CAS-induced IgG1 and IgE antibody responses. **(a) Reduction of DC migration in the dLN of *Cd3e*-deficient mice.** The dLN 7 acells were obtained from CTRL (n=5) and *Cd3e*-deficient (CD3e KO) mice (n=9) treated with CAS (D17). DC populations were evaluated as described in Fig. 4d. Data show the mean ± SD of the percentage of total CD11c^+^, CX3CR1^+^MHCII^+^, CD11c^hi^MHCII^dull,^ and CD11c^dull^MHCII^hi^ DCs. (**b**) **Th2 and T**_**FH**_ **development under the *Cd28*-deficient condition.** Th2 and T_FH_ profiles and memory status were assessed as described in Fig.6c in the LNs from the CAS-treated mice on D17 CTRL (n=6) and *Cd28*-deficient (CD28KO) (n=5) mice, Data show the mean ± SD of the cell number. (**c**) **DC migration under the *Cd28*-deficient condition.** Spleen cells were obtained from CTRL (n=5) and CD28 KO mice (n=3) treated with CAS+NP-OVA (D31). Cells were stained for CX3CR1, MHCII, CD11b, CD11c, CD24, and CD172a in the linage^−^ cells. The left shows representative flow cytometry. The right panel shows the mean ± SD of the percentage of CD11c^hi^MHCII^dull^ and CX3CR1^+^MHCII^+^ in CD11c^+^ cells. (**d**) **IgG1 and IgE responses in Il13-deficient Th2 transferred mice** The left panel shows the experimental design. Th2 cells were generated from CD45.2^+^ OTII-IL-4/hCD2 mice crossed with *Il13* -sufficient (OTII Th2) and -deficient (IL-13KO OTII Th2) mice. The purified IL-4^+^ Th2 cells were transferred into *CD28*^−*/*−^ mice. The transferred mice were immunized with OVA in alum and analyzed at D6. The right panel shows mean ± SD of serum titers of anti-NP29 and anti-NP1 IgG1 and IgE in the mice receiving OTII Th2 (n=5) or IL-13KO OTII Th2 (n=5) cells. P values by unpaired *t*-test are indicated as * p<0.05, ** p<0.01, *** p<0.001, **** p<0.0001 with unpaired *t*-test.

**Figure 8.**
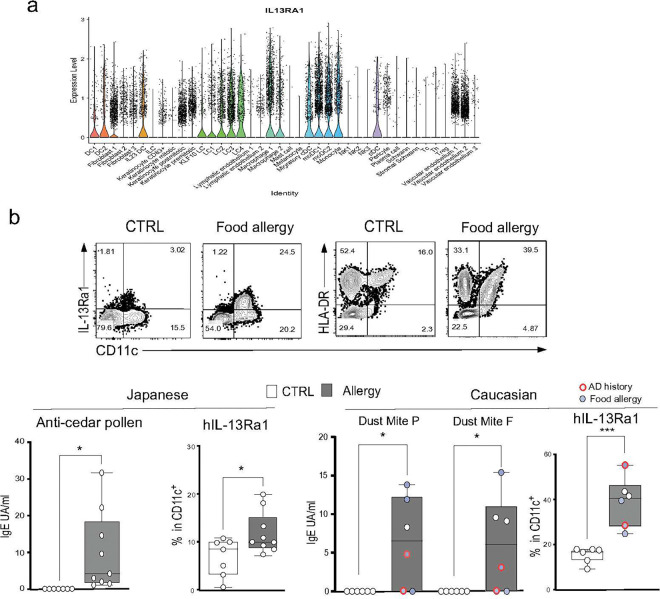
Expression of IL-13R in human PBDCs **(a) IL-13Ra1 expression in healthy human skin DCs.** IL-13Ra1 expression was analyzed in the cohort study for human skin.^49^ The figure shows violin plots of IL-13Ra1 in various cell types in the skin. (**b**) **The expression of IL-13Ra1 and MHCII in Japanese and Caucasian individuals.** The upper panel shows flow cytometry data (hIL-13Ra1 (left) and HLA-DR (right)) of PBMCs from Japanese subjects, seven healthy controls and nine subjects with cedar pollen (CP) allergic rhinitis who had IgE antibodies specific for CP. The lower left panel indicates mean ± SD of the anti-CP IgE titers and percent h IL-13Ra1 positive cells in the linage^−^(CD3, B220) CD11c^+^ DC population. The serum samples were from seven controls (white) and nine allergic (gray) individuals. The lower right panel indicates PBMCs from Caucasian subjects, six with IgE antibodies specific for food and dust mite allergens, and six healthy controls. The Caucasian subjects included two active AD (red line circles) and four food allergy individuals (blue circles). The figure indicates the mean ± SD of the anti-dust mite IgE titers and percent positive cells of hIL-13Ra1. P values are indicated as * p<0.05, ** p<0.01 of unpaired *t*-test.

The effect of the IL-13 signal was also observed in the splenic DCs after treatment with CAS+NP-OVA (Fig.5c **S-Fig.7**). The IL-13Ra1^ΔDC^ mice showed a significant reduction in the CD11c^hi^ DCs but not in the CD11c^dull&lo^ DCs despite no change in the MHCII MFI. As shown in Fig.4c, the fraction of CD11c^dull&lo^ DCs contained PBDC-derived CX3CR1^+^DC, and consistently, the IL-13Ra1^ΔDC^ PBDC showed no change in the proportion of the MHC^+^DCs, which displayed CD11c^dull^ (**S-Fig.8**). These results indicated that IL-13 signal was defective only in CD11c^hi^DCs of the IL-13Ra1^ΔDC^ mice. These defective DCs predominantly expressed CD172a, the marker of cCD2s, and co-expressed costimulatory molecules, such as CD301b, PDL2, CD86, CXCR5, and ICOSL.^45^ The expression of CD301b, PDL2, and CD86 was increased in control CD11c^hi^ DCs, while the cell number and the proportion of the CD11c^hi^DCs, but not in the CD11c^dull^DCs, expressing these costimulation molecules were significantly reduced in IL-13R^ΔDC^ mice (Fig.5c **S-Fig.7**). Previous literature reported that CD301b is a marker for the cDC2s directing Th2 differentiation.^46^ Therefore, we speculated that the role of the IL-13 signal in DCs is to control cDC2 functions, thereby promoting efficient type 2 humoral responses.

Next, we investigated the role of IL-13 signaling in DC function in the dLN following the CAS treatment. In the cervical LNs near the CAS-treated site, the control mice exhibited two major populations, CD11c^hi^MHC^dull^ DCs, and CD11c^dull^MHC^hi^ DCs (Fig.5d). CX3CR1 was highly expressed in CD11c^hi^ DCs but not in CD11c^dull^ DCs. The IL-13Ra1^ΔDC^ mice showed a marked reduction of the CD11c^hi^ DCs. The IL-13Ra1^ΔDC^ mice also exhibited reduced expression of MHCII, CD301b, PD-L2, and CD86 in CD11c^hi^MHC^dull^ DCs but not in CD11c^dull^MHC^hi^ DCs (Fig.5d, **S-Fig.8**). Therefore, it is reasonable to conclude that the IL-13 signal contributes to the accumulation of migratory CX3CR1^+^DCs in the LN localized close to the skin sensitization site.

Previous reports indicated that CX3CR1 promotes the development of myeloid precursors into DCs under steady-state conditions.^47^ Thus, it is likely that CX3CR1^+^ PBDCs would be a possible precursor of cDC2s.To investigate this possibility, we isolated CX3CR1^+^PBDCs and cultured them with IL-4 or IL-13 (Fig.5d, **S-Fig.6**). IL-4 enhanced the number of MHC^+^DCs, while IL-13 expanded more MHCII^hi^DCs compared to IL-4. Moreover, IL-13 significantly increased the expression of ICOSL by CD301b^+^MHCII^hi^DCs (Fig.5d). These MHCII^hi^ DCs contained CD24^dull^CD172a^+^ cells, indicating that most of the cells were cDC2s. Taken together, these *in vivo* and *in vitro* data suggest that the IL-13 signal in DCs promoted the maturation of cDC2s, directing Th2 differentiation by enhancing the expression of MHCII, CD301b, CD86, PD-L2, and ICOSL. The increased expression of MHCII and CD86 in CD301b^+^ cDC2s was thought to enhance antigen-presenting ability, promoting T cell activation in type 2 humoral immunity.

### The IL-13 signal in cDC2s impacts the generation of IL-4-producing T_FH_ cells and memory T cells.

The results in Fig.5d demonstrated that the IL-13 signal affects the ability of CD11c^hi^cDC2s to function as antigen-presenting cells even in the cervical LN after CAS treatment. Moreover, the anti-OVA IgG1 and IgE responses induced by CAS treatment alone were significantly lower in the IL-13R^ΔDC^ mice than in the control (**S-Fig.9**). Therefore, we speculated that the defect of the IL-13 signal affects primary T cell activation. To understand which CD4 T cell subsets are activated in the dLNs and the spleen of mice treated with CAS and CAS+NP-OVA, we examined changes in the CD4 T cell subsets by CyTOF analysis. The t-SNE projection indicated T_FH_ /memory T (Tm)(CD27^+^PD-1^hi^), central Tm (Tcm) (CD44^hi^CD62L^hi^CD27^+^), and PD-L1^hi^ CD4 T cells subsets were increased in the CAS treated dLNs (Fig.6a, **S-Fig.10**). On the other hand, the CAS+NP-OVA treated spleen exhibited an increase of T_FH_ /Tm, effector T (Te)(CD27^−^PD-1^dull^), and CCR7^+^T subsets. These results indicated that skin sensitization by CAS treatment actively enriches T_FH_ and memory CD4 T cells.

To confirm the role of the IL-13 signal in primary and secondary CD4 T cell activation, naïve CD4 T cells obtained from the OTII mice crossed with hCD2 IL-4 reporter (IL-4/hCD2) mice were transferred into control or IL-13R^ΔDC^ mice to track the expression of IL-4/hCD2 in Th2 (CXCR5^−^) and T_FH_ (CXCR5^+^) cells (Fig.6b). The number of IL-4^+^ T_FH_ cells and Th2 cells decreased in the dLN following CAS treatment, and a similar reduction of IL-4^+^ T_FH_ cells was also found in the spleen and dLN after the secondary priming on D31. Along with the decline in T_FH_ cells, the IL-13R^ΔDC^ recipient mice showed a significant reduction in the number of GL-7^+^GC-B cells in the dLNs on D17 and D31 and in the spleen on D31 (Fig.6b). The reductions were prominent among host Th2 and T_FH_ cells compared to the transferred OTII T cells (Fig.6c, **S-Fig.11**). CAS treatment upregulated several Th2 markers, CCR4 and ST2/IL-33R, and the T_FH_ markers, ICOS, and IL-13R^ΔDC^ mice showed prominent reductions of these makers. Previous studies have shown that MC903-induced TSLP expression supports Th2 and T_FH_ differentiation in several mouse models.^16, 48^ Consistent with these findings, IL-13R^ΔDC^ mice had a prominent reduction of TSLPR expression in the host CD4 T cells (Fig.6c). We also observed a reduction in CD27^+^ central memory (Tcm)(CD62L^hi^CD44^hi^) and effector memory (Tem) (CD62L^−^CD44^hi^) cells (Fig.6c). Furthermore, scRNAseq analysis indicated the transferred OVA-specific OTII T cells differentiated into two major clusters: C0 and C1 on D31 of the spleen (Fig.6d). The C1 island contained T_FH_ signature genes (*Bcl6*, *Cxcr5*, and *Icos*), while the C0 island had non-T_FH_ signature genes (*Ccr7*, *S1pr1* and *Gata3*). Interestingly, the expression of the T_H_2 master regulator Gata3 was more abundant in C1 than C0, indicating that the transferred OTII T cells had differentiated into T_FH_ cells expressing GATA-3 and IL-13, corresponding to T_FH_2 or T_FH_13 cells (Fig.6d).^1, 2^ The distribution pattern of the Bcl6-, Gata3-, and Icos-expressing OTII T cells was less abundant in C1 of IL-13R^ΔDC^ mice compared to the control mice. IL-13 expressing T_FH_2 cells were not present in the IL-13R^ΔDC^ mice (**S-Fig.12**). These results indicate that the DC-derived IL-13 signal is critical for generating the T_FH_2 cells co-expressing IL-4 and IL-13, both of which are required for generating high-affinity IgE Abs.

### T cell-derived IL-13 controls the fine-tuning antigen-presenting ability of cDC2s.

Previous literature has reported that skin ILC2-derived IL-13 in steady-state facilitates the antigen-presenting ability of CD11b^dull^migDC2s.^22^ Indeed, a decrease in MHCII expression was observed in the splenic DCs of IL-13Ra1^ΔDC^ mice (Fig.5b). To investigate whether the ILC2-derived IL-13 contributes to the migration of DCs into dLNs in the CAS-induced mouse, we treated *Cd3e* deficient mice with CAS and on D17 assessed the number of CD11c^hi^MHC^dull^ DC subset cells in the dLN, which contained a large number of CX3CR1^+^DCs (Fig.7a, **S-Fig.13**). The loss of T cells induced a striking reduction in the attraction of two major mature DC subsets, CD11c^hi^MHC^dull^ DC and CD11c^dull^MHC^hi^ DCs even though the total number of DC in the dLN was unchanged. These results suggested that T cell-derived IL-13 might be required for the initial maturation of the DCs localizing in the dLN near the skin sensitization site.

Next, we hypothesized that the OVA-specific memory Th2 cells generated by CAS treatment could become the IL-13 source in the secondary response. To test this hypothesis, we used CD28-deficient mice, which were impaired in memory formation due to the lack of initial T cell activation (Fig.7b). CAS-treated CD28-deficient mice exhibited a striking reduction in the number of CD4 T cells expressing pathogenic markers, CCR4, ST2, and the number of Tcm and Tem cells was also markedly reduced in the dLNs on D17 (Fig.7b). Under this condition where Tcm and Tem cells were less dominant, the spleen of the CD28-deficient mice additionally immunized with NP-OVA on D31 showed significantly fewer CX3CR1^+^DCs and CD11c^hi^MHCII^dull^ DCs, which also expressed the cDC2 marker, CD172a (Fig.7c). Therefore, these results suggest that the OVA-specific Tm cells could be the primary source of IL-13 required for the maturation of CD11c^hi^MHCII^dull^cDC2 in the spleen after systemic immunization.

We recently reported that Th2 cells further differentiate into IL-13-producing T_FH_2 cells after secondary antigen stimulation.^2^ Similar T_FH_2 cells were also generated in the spleen of mice treated with CAS+NP-OVA (Fig.1b), suggesting that memory Th2 cells could be a source of the IL-13 required for cDC2 maturation regulating T_FH_2 generation. Therefore, we investigated the role of IL-13 from Th2 cells in the IgG1 and IgE responses using adoptive transfer of *ex vivo* generated Th2 cells (Fig.7d). The transfer of OTII-derived Th2 cells lacking IL-13 resulted in significantly lower titers of anti-NP IgE and high-affinity IgE, but not IgG1, than in mice transferred with IL13-sufficient Th2 cells (Fig.7e). These results indicated that memory Th2 cells-derived IL-13 controlled cDC2 maturation required for the IgE and high-affinity IgE responses.

### Human L-13Ra1 on PBDC is a marker of AD history

Fig.4c indicated that CX3CR1^+^PBDCs increased the expression of IL-13Ra1 and MHCII following CAS+NP-OVA treatment, which mimics secondary allergen sensitization in human AD patients. Therefore, we assumed that IL-13Ra1 and MHCII expression on PBDCs would be markers for the CAS-mediated activation. The expression of IL-13Ra1 in skin DCs has also been demonstrated in a recent large-scale cohort study, and IL-13Ra1 has been shown to be expressed constitutively by migratory cDC of healthy human skin (Fig.8a).^49^ We therefore conducted further analysis to investigate the correlation between IL-13Ra1 and MHCII in PBDCs newly isolated from human allergy patients. The Japanese subjects included nine with allergic rhinitis to cedar pollen (CP) who had IgE antibodies specific for CP, and six negative controls for analysis during the pollen non-dispersal period. The Caucasian subjects included six with IgE antibodies specific for food and dust mite allergens and six negative controls. This group also included two with active AD and four with food allergies. We found a significant increase in the percentage of IL-13Ra1^+^DCs in both Japanese and Caucasians (Fig.8b), although the trend toward increased expression of IL-13Ra1 was more prominent in the Caucasian group. All six individuals also showed an increased number of MHCII^hi^ (HLA-DR) DCs (Fig.8b). Therefore, consistent with the mouse CAS model, the expression of IL-13Ra1 and MHCII by circulating DCs correlated with the CAS and AD history. These results demonstrated that the IL-13Ra1^+^DCs may circulate in the bloodstream of individuals with AD history. Therefore, IL-13Ra1 and MHCII expression on PBDCs should be good markers for AD history and predict susceptibility to future allergic march.

## Discussion

The present work indicated that CAS confers a specific type 2 microenvironment that promotes high-affinity IgE responses via the IL-13-mediated activation of cDC2s. In the primary CAS response, the IL-13 signal is critical in promoting Th2 memory and T_FH_ responses by accumulating CX3CR1^+^ migratory DCs in dLNs. In the secondary response, CX3CR1^+^ PMDCs were necessary for allergen delivery into distal lymphoid tissue to induce IL-13 production by memory Th2 cells. The Th2-derived IL-13 plays a role in expanding the MHCII^+^cDC2s to confer better antigen presentation capability. The IL-13 signals also enhanced the expression of CD301b, PD-L2, and CD86 by the MHCII^+^cDC2s, accelerating the differentiation of T_FH_2 cells and subsequently enhancing the GC-dependent allergen-specific high-affinity IgE response. Therefore, a serial combination of IL-13 signals and CX3CR1 expression by the DCs are central in connecting skin and distal lymphoid organs in a continuum.

The first studies of IL-13 loss-of-function mice indicated a subtle effect on Th2 cytokines and a robust impact on IgE but not on other isotype responses.^30^ Loss-of-function and blocking studies also suggest that IL-13 signaling appears involved in IgE responses induced primarily by antigen exposure through the airways and skin but not in systemic responses.^29, 30, 31^ Previous reports proposed that T_FH_13-derived IL-13 independently controls high-affinity IgE antibody production via sequential class-switching in the GC-B cells.^38^ However, the present study demonstrates that IL-13Ra1 expressing DCs, especially cDC2s, are a significant target in the IgE responses against the allergen. Interestingly, the IL-13-dependent pathway was selectively active only when the initial allergen was introduced from damaged skin.

It has been reported that IL-13 is critical for the functional modification of dermal DCs to promote Th2 generation and attraction in allergen protease-induced asthmatic inflammation.^19, 20, 21, 50^ A previous study suggested that the skin resident CD11b^mid^ cDC2s may be a target of IL-13 signaling at steady state.^22^ In this case, homeostatic IL-13 from dermal ILC2s regulates the population size of CD11b^mid^ cDC2s to promote Th2 differentiation. However, the CyTOF data in Fig.3b indicated that CD11b^hi^DCs (C12) would be skin resident DCs, while the CD11b^mid^ skin DCs (C15) highly expressed the fractalkine receptor CX3CR1 responsible for attracting DCs to the lymphatic endothelium to establish type 2 microenvironments in the dLN.^51, 52^ Since the CX3CR1^+^ DCs were abundant in PBMC (**S-Fig.6**), they were the population circulating in the blood and lymphatic vessels. Interestingly, both skin DC populations expressed IL13Ra1 and significantly increased in the cervical LN after CAS (Fig.4a). The CX3CR1^+^ DCs were CD11c^hi^ in the dLNs around the CAS site. They were significantly reduced in the absence of the IL-13 signal (Fig.5d), suggesting antigen delivery to dLNs by the skin DCs was IL-13-dependent under the CAS condition. This observation was consistent with the global view of the immune cell responses to 86 cytokines, suggesting that cDCs and migDC are primary functional targets of IL-13 in mouse LNs *in vivo*.^53^ The CD301b^+^ DCs within the subepithelial regions in the skin are reported to be responsible for transporting cutaneous antigens to the dLN.^46^ Our *in vivo* results demonstrated that loss of the IL-13 signal in the DCs reduced the number of CD301b^+^DCs, indicating the contribution of the IL-13 signal to the expression of CD301b by the CX3CR1^+^migDCs. Previous reports indicated that IL-13 enhances Th2 differentiation via the interaction between antigen-loaded migDC2s and CD4^+^ T cells.^54^ Recently, “macro-clustering” of CD301b^+^ migDC2s has been reported to promote and specialize Th2 cell development.^55^ Therefore, the IL-13 signal contributes significantly to the accumulation of CX3CR1^+^ DCs and expression of MHCII and CD301b, directing to a robust Th2 response, and then some of the Th2 cells could remain and circulate as memory Th2 cells.

IL-13 signaling in DCs is also associated with high-affinity IgE responses in secondary systemic responses. However, unlike the CAS-mediated response, the target of IL-13 signaling would be CX3CR1^−^cDC2s, which constitute a significant CD11c^hi^ population in the spleen. Because they also express IL-13Ra, IL-13 influenced the CX3CR1^−^cDC2s to increase the expression of MHCII, CD301b, PD-L2, and CD86 (Fig.4c & Fig.5c). This is consistent with the in vitro culture. IL-13 selectively promoted the maturation process of cDC2, which expressed high levels of MHCII, CD301b, and ICOSL (Fig.5d). The effect of IL-13 was more pronounced than that of IL-4. These results suggest that IL-13 signaling is a potent driver of cDC2s to promote type 2 T cell responses. This observation raises the possibility that some cDC2s are derived from CX3CR1^+^PBDCs. Therefore, in the secondary responses, the IL-13 signal acted mainly on the cDC2 population, accelerating the expression of MHCII and several costimulatory molecules that drive the T_FH_2 response. Moreover, IL-13 signaling was also required to modify the immunological characteristics of patrolling CX3CR1^+^PBDCs into cDC2s. Another important mechanism in secondary responses was the role of CX3CR1^+^DC, which is required for antigen delivery to Th2 memory cells in the spleen. The CX3CR1-CX3CL1 interaction may allow the CX3CR1^+^PBDCs to enter the inflamed spleen, where the blood vessel endothelium expresses CX3CL1.^(51,54)^ A previous publication showed that CX3CL1-neutralizing antibodies inhibit the migration of allergen-loaded DCs to lymph nodes and inflammatory sites.^(52)^ Blocking CX3CR1^+^ DC entry into the spleen with the CX3CR1 antagonist JMS-17–2 abolished anti-NP IgG1 and IgE responses after systemic immunization with NP-OVA (Fig. 4d), supporting the idea that CX3CR1 expression may be a critical mechanism in controlling the influx of patrolling CX3CR1^+^ DCs into the inflamed spleen.

In secondary responses, memory Th2 cells would be the initial source of IL-13, driving activation of cDC2 in the spleen because *in vivo* transfer of OVA-specific Th2 cells generated high-affinity anti-NP IgE Abs after NP-OVA second priming, whereas the transfer of IL-13-deficient Th2 impaired these IgE responses (Fig.7d). CD28-deficient mice showed a lack of IgE responses along with a significant reduction of cDC2s (CD11c^hi^ MHCII^dull^) in the spleen (Fig.7c). These results suggest that memory Th2-derived IL-13 is essential for the activation of cDC2s required for the generation of allergen-specific high-affinity IgE Abs. More interestingly, CAS+NP-OVA selectively generated the IL-13-expressing T_FH_2 cells (Fig.1b). However, the OTII T cells transferred into IL-13Ra1^ΔDC^ recipient mice had less IL-13 expression in the T_FH_ cluster (**S-Fig.10**). Therefore, in a later phase, the Th2-derived T_FH_2 would be an alternative source of IL-13 to accelerate MHCII expression on cDC2s in a paracrine manner.^2^ On the other hand, the source of IL-13 involved in the initial DC activation of the dLN immediately after CAS treatment appears to be mechanistically different. In CD3ε-deficient mice, the accumulation of DCs in the dLN was drastically reduced (Fig.7a), suggesting that T cell-mediated IL-13 may also be required for traffic between the skin and the LN, but how the initial trigger of Th2 is introduced remains unclear. Therefore, the exact mechanism by which Th2-derived IL-13 regulates DC migration requires further investigation.

The CAS concept has also been found to be the case in a human cohort study showing that CAS in the context of AD, but not in the non-AD context, increases the risk of asthma as part of the atopic march.^(64–66)^ Consistently, type 2 inflammation increased the number of IL-13R+ PBDCs in humans and mice (Fig.4c & Fig.8b). Thus, the increased expression of MHCII and IL-13Ra1 are excellent markers to trace whether initial antigen priming is introduced via the skin. These results suggest that initial skin antigen sensitization efficiently dictates type 2 immune properties in distal lymphoid organs via circulating memory Th2 cells and the attraction of the IL-13R^+^ DCs.

In conclusion, the present study proposes the importance of IL-13 signaling in connecting the skin barrier and the secondary lymphoid organs. In the context of skin sensitization, dermal DCs and/or CX3CR1 DCs play a role in allergen delivery to LNs and spleen, and IL-13 has a selective role in providing the activation signal for cDC2s to increase antigen-presenting capacity and promote robust IgE responses via memory Th2 and T_FH_2 cells. IL-13-dependent cDC2 activation induced by increased allergen penetration into the skin is tightly linked to allergen-specific pathogenic IgE responses. Therefore, IL-13R DCs could be reasonable therapeutic targets in IgE-mediated allergic responses and in controlling the atopic march.

## Material and Methods

### Mice

The mouse strains used in these experiments, C57BL/6J, OTII, and IL-4-hCD2Bac Tg mice, are described in previous reports.^28^ IL-13-tomato mice were kindly provided by Andrew Mackenzie (MRC Laboratory of Molecular Biology).^37^ *Icos*^−/−^ mice were kindly provided by Japan Tobacco Inc. (Tokyo, Japan).^56^ *Cd4-cre and Cd79a-cre* mice are described in previous reports,^57^ and *Cd11c-cre* mice were a gift from Boris Reizis (Columbia University).^58^ *Il13ra1* floxed mice were generated using the CRISPR/Cas9 system (Cyagen Santa Clara, CA. USA). Targeting contracts using a self-deletion anchor-containing neo cassette and the primers used for construction and genotyping are described in **S-Fig.1** and **S-Table 1**. Targeted ES cell clones were injected into C57BL/6 albino embryos, which were then re-implanted into CD-1 pseudo-pregnant females. Founders were identified by their coat color and germline transmission was confirmed by breeding with C57BL/6J females.

All experiments were conducted with the approval of the Tokyo University of Science and RIKEN IMS Institutional Animal Care and Use Committees. Animals were housed on a standard 12:12 hour light-dark cycle with free access to food and water. All mice used in this study were adults (8–12 weeks) and were maintained under specific pathogen-free conditions. Mice were either randomly assigned to treatment groups or assigned to groups based on genotype. Experiments were performed on independent cohorts of male and female mice. No differences between sexes were observed and no analyses of the influence of sex were performed.

### Human subjects and isolation of cells

Nonallergic healthy volunteers and volunteers with seasonal allergy symptoms around the time of seasonal pollen dispersal were recruited at the Department of Pulmonary Medicine, Kagoshima University. Healthy subjects with no nasal clinical history and no IgE for specific Japanese cedar pollen, cypress pollen, house dust mites, orchard grass pollen, or ragweed pollen were included as healthy controls. Subjects in the Japanese cedar pollen-induced allergic rhinitis group had typical nasal symptoms during the cedar pollen season and elevated IgE specific for Japanese cedar pollen (≥0.70 UA/mL). All human experimental procedures were reviewed and approved by the Ethics Committee on Clinical Research, Sakuragaoka Campus, Kagoshima University.

Caucasian blood samples were obtained from participants in the Benaroya Research Institute Registry and Repository. This study was approved by the Benaroya Research Institute Institutional Review Board (IRB) (Protocol IRB07109–431). The patients with allergies were age- and sex-matched with healthy controls. All PBMC samples were obtained under approved research protocols with informed consent. PBMCs were isolated by diluting the blood 1:1 in PBS and were prepared by density-gradient centrifugation according to standard protocols.

### DC and skin cell preparations

For DC preparation, spleen and dLNs were collected, cut into small pieces, and digested in 10% FBS in RPMI containing 100mg/ml Liberase TL (Roche) and 100mg/ml DNase I (Roche) for 30min at 37°C and 200rpm in a shaking incubator. Skin cells were prepared from the ears using the gentleMACS^™^ Octo Dissociator (Miltenyi Biotec) in the RPMI solution containing 250 mg/ml Liberase TL (Roche) and 50mg/ml DNase I (Roche). Single cells were isolated using a cell strainer.

### CAS mouse model and active cutaneous anaphylaxis (ACA)

For the CAS treatment, the bilateral ear skin was treated topically with MC903 (2mM in 100% ethanol) and OVA (100 μg, grade V; Sigma, St. Louis, MO) (100μg in 20 μL of PBS) daily for ten days to sensitize the mice. Unsensitized control mice were treated with vehicle (100% ethanol). For systemic sensitization, mice were given an i.p. injection of an NP16-OVA conjugate (100μg in PBS) and alum adjuvant (FUJIFILM Wako Pure Chemical Co., Tokyo, Japan) (9 μg) on day 26. For ACA analysis, mice were subcutaneously challenged with NP16-OVA on day 56, and rectal temperature was measured every 5 minutes to assess anaphylaxis.

### Flow cytometry and immunohistological staining

Mouse and human cells were stained with the reagents for flow cytometry staining listed in **S-Table 2**. Flow cytometry was performed on a FACSCalibur and FACS Melody ^18^, and data were analyzed using FlowJo (Tree Star, CA, USA). For immunohistological staining, tissue sections were fixed with 4% PFA overnight at 4°C and then penetrated with a 10% sucrose solution. Fixed tissue was frozen in OCT compound (4583, Sakura Finetek, Japan), and 8-μm sections were prepared by cryostat (Leica, Wetzlar, Germany) and fixed onto slide grasses. After permeabilization (0.1% Triton X-100) and blocking (3% BSA-PBS), the sections were stained with the reagents for immunohistological staining listed in **S-Table 2**. Images were acquired using a fluorescence microscope BZ-X710 (Keyence, Osaka, Japan).

### ELISA and antibody affinity assay

OVA- or NP-specific IgG1 and IgE concentrations were measured by ELISA using the following protocols. Plates were coated with OVA (100μg/ml), NP29-BSA (10μg/ml), or NP1-BSA (10μg/ml for IgG1, 0.5μg/ml for IgE) in 0.1M carbonate-bicarbonate buffer overnight at 4°C. After washing, 5% BSA in PBS was used to block nonspecific binding, and serum samples were added and incubated for 1 hour at 37°C. The following is the list of secondary antibodies: Anti-IgG1-HRP (A90–105p; BETHYL), anti-IgE-biotin (553419; BD), and StAv-HRP (43–4323; Invitrogen). BD OptEIA (555214, BD) was used for detection. Anti-OVA IgE was measured with a Mouse IgE ELISA OVA kit (DS Pharma Biomedical) according to the manufacturer’s protocol.^2^

### Mass Cytometry by time of flight (CyTOF) analysis

Spleen, dLNs, skin cells, and PBMC were prepared from NS, CAS, and CAS+NP-OVA-treated mice. Cells (3×10^6^) were stained with 33 metal-conjugated antibodies (S-Table 3), Maxpar OnDemand Mouse Immune Profiling Panel Kit (Standard Biotools Cat:9200001) in a final volume of 100μl Maxper Cell Staining Buffer. Anti-IL-13R Ab was labeled with 154Sm by a Maxpar^®^ X8 Antibody Labeling Kit (201154A). Cells were incubated for 30 min at room temperature with 1uM Rhodium (Standard Biotools Cat:201103A) and 5 μl of FcR Block (Biolegend Cat:422301) to exclude dead cells and nonspecific binding of antibodies. Cells were washed twice with Maxper Cell Staining Buffer and were resuspended in 1ml of 1.6 % PFA (Thermo Fisher Scientific Cat: 28906) in PBS and then incubated at RT for 10min. Cells were acquired on a CyTOF mass cytometer to Helios specifications (CyTOF/Heslious) (Standard Biotools). 0.1× EQ Four Element Calibration Beads (Fluidigm) were added to the samples for data normalization of the FCS files using the CyTOF software. A total of 100,000 events were recorded for each sample and analyzed on Cytobank.

In the data analysis, positive events were first identified and gated. Then, non-viable cells and equalization beads were excluded to isolate live cells using FlowJo software (version 10.4.2). For clustering DC subsets and CD4 T cell subsets, CD3^−^ CD19^−^ CD11c^+^ MHC-II^+^ F4/80^−^ Ly6C^−^ cells and CD3^+^ CD19^−^ TCRb^+^ TCRgd^−^ CD4^+^ CD8^−^ cells were defined as DC and CD4 T cells respectively. We then down-sampled the live cells to 1000 cells per sample and down-sampled the DC and CD4 T cell populations to 500 cells each. These down-sampled files were then loaded into the R Catalyst package (version 1.22.0) and integrated with metadata. Subsequently, we performed FlowSOM (Flow Self-Organizing Maps) clustering using the following parameters: Features set to “type”, the mapping dimensions at x.dim=10 and y.dim=10, seed value “1234”, and the maximum number of clusters (max.k) set at 20 for live cells and 10 for DC and CD4 T cells. Following the FlowSOM clustering, we employed t-Distributed Stochastic Neighbor Embedding (tSNE) to visually represent our data, utilizing the runDR and plotDR functions.

### Single-cell RNA-seq library preparation, sequencing, and data analysis.

According to a previous report, the data for the DC scRNAseq was generated by BD Rhapsody TAS-Seq.^59^ Briefly, cells were stained by BD sample tag (BD Biosciences), and CD11c^+^ MHCII^+^ Lin^−^ (CD3, B220, NK1.1, Ly-6G, TER119) splenic cells were sorted using a BD FACS Melody Cell Sorter. Then, 20,000 live cells were loaded onto the BD Rhapsody cartridge; cDNA trap and amplification were performed using the TAS-Seq method. Sequencing was performed with an Illumina Novaseq 6000 sequencer (Illumina, San Diego, CA, USA) according to the manufacturer’s instructions (Read1: 56 base-pair, read 2: 155 base-pair). The pooled library concentration was adjusted to 1.75 nM, and 1% PhiX control library v3 (Illumina) was spiked into the library. Pair-end Fastq files (R1: cell barcode reads, R2: RNA reads) were processed as follows. Adapter trimming, quality filtering, and phase-shift base removal of sequencing data were performed by using Cutadapt 4.1(https://doi.org/10.14806/ej.17.1.200). Filtered cell barcode and cDNA reads were annotated and mapped to a reference genome (build GRCm39 release-107) by using STARsolo (2.7.10a)^60^ and known BD Rhapsody cell barcodes by the following parameters: –outSAMmultNmax 1–outFilterScoreMinOverLread 0 –outFilterMatchNminOverLread 0–outFilterMultimapScoreRange 0 –seedSearchStartLmax 30–soloType CB_UMI_Complex –soloUMIdedup NoDedup –soloMultiMappers Rescue –soloFeatures Gene GeneFull – soloCBmatchWLtype EditDist_2 –soloUMIlen 8 –soloCBposition 0_0_0_8 0_13_0_21 0_26_0_34 –soloUMIposition 0_35_0_42. Results from non-umi count data were converted to genes x cells matrix files, and the inflection threshold of the barcode rank plot was detected by using the DropletUtils package in R version 4.2.1.^61, 62^ Valid cell barcodes, and backgrounds from knee-point threshold to inflection threshold * 1.1 were separated by the Dropkick package (https://github.com/KenLauLab/dropkick).^63^ Associated sample tag reads were mapped to known barcode fasta by using bowtie2–2.4.2^64^ by the following parameters: -p 2 -D 20 -R 3 -N 0 -L 14 -i S,1,0.75 –norc –seed 656565 –reorder –trim-to 3:40 –score-min L,−9,0 –mp 3,3 –np 3 –rdg 3,3. Then, the cell barcode information of each read was added to the bowtie2-mapped BAM files and read counts of each tag in each cell barcode were counted using mawk. Results from the count data were converted to Tags x cells matrix file using the “data. Table” package in R version 4.2.1. For the assignment of each tag to each cell barcode, read counts of each tag in each valid cell barcode, which are defined by the cDNA matrix, were extracted from the tag/cell barcode expression matrix. Unassigned cell barcodes were labeled as “not-detected” cells. Then, a sum of the total read counts of each tag was normalized to 10M reads and the log2 fold-change between the first most counted tags and the second most counted tags within each cell barcode. Doublet cells (double-positive cells of any pair of Tags) were identified by the flowDensity package; (https://www.bioconductor.org/packages/release/bioc/html/flowDensity.html) and log2 fold-change between the first and second most counted tags under 0.30906 were identified as doublets. Finally, the remaining cell barcodes were assigned to the first most counted tags. Results from the tag-assigned count matrix file were processed through the R package Seurat 4.1.0.^65^ We first created a Seurat object for each dataset using the CreateSeuratObject function. We then filtered out mitochondrial gene-high cells (over 25%) and performed global normalization using the normalizeData function (scale.factor = 1,000,000). Next, highly variable genes of each dataset were identified using the FindVariableFeatures function (selection.method=mvp, mean.cutoff=c (0.1, Inf), dispersion.cutoff=c(0.5, Inf)). PCA analysis was performed based on the 8983 highly variable genes identified by the FindVariableFeatures function, and the data were scaled using the ScaleData function. Next, the enrichment of each PC was calculated using the JackStraw function, and statistically significant PCs (p≤1×10^−5^) were selected for clustering and dimensional reduction analyses. After performing linear dimensional reduction using PCA by the RunPCA function, the cells were clustered by the FindNeighbors function (dims=1:69) and the FindClusters function (resolution=0.6). Then, we performed linear dimensional reduction using Uniform Manifold Approximation and Projection (UMAP) by the RunUMAP function. Statistically significant marker genes between two groups were identified using the FindMarkers function (min.pct=0.25). Cell type estimation was performed using the SingleR package.^66^

For scRNAseq of T cells, PI^−^ Vb5.1^−^5.2^+^ CD4^+^ splenic OTII T cells were sorted into 96 well plates using a FACS Melody^18^ and then lysed in a buffer containing dNTPs and oligo(dT)-tailed oligonucleotides with a universal 5’-anchor sequence. A reverse transcription reaction, which adds 2–5 untemplated nucleotides to the cDNA 3’ end, was then performed. A template-switching oligo carrying 2 riboguanosines and a modified guanosine was added to produce an LNA as the last base at the 3’ end. After the first-strand reaction, the cDNA was amplified using 25 cycles. Next, Tn5 tagmentation was used to construct sequencing libraries from the amplified cDNA (Nextera XT DNA Library Preparation Kit, Illumina). The sequencing was carried out using NovaSeq6000 equipment (150PE, Illumina). Gene expression obtained in scRNAseq experiments was counted using the featurecounts (version 2.0.1) and an mRNA database based on the mouse RefSeq reference (mm 10). Then, we created a Seurat object from each gene expression matrix using CreateSeuratObject function. We normalized mRNA expression with log-normalize using normalizeData function (scale. factor = 10,000). Then, the top 2,000 most highly variable genes were identified by using FindVariableFeatures function. After scaling the data by ScaleData function, we performed linear dimensional reduction using PCA by RunPCA function. Then, the cells were clustered by FindNeighbors function (1:30) and FindClusters function (resolution=0.8). We performed linear dimensional reduction using UMAP. Statistically significant marker genes between two groups were identified using the FindMarkers function (min.pct=0.25).

### Quantification and statistical analysis.

Distributed group data were expressed as mean ± standard deviation (SD) and statistical significance was determined using the two-tailed Student’s t-test. Data were analyzed with GraphPad Prism 7 software (GraphPad Software, La Jolla, CA, USA). All data in the present works were normally distributed. Differences were recognized as significant with a p-value of <0.05.

## Data Availability

Single-cell RNA-seq data will be uploaded to GEO and made publicly available when this manuscript is accepted for publication.
